# Genetic Variants and Clinical Features of Patients With Glycogen Storage Disease Type Ib

**DOI:** 10.1001/jamanetworkopen.2024.61888

**Published:** 2025-02-26

**Authors:** Yu Xia, Yu Sun, Taozi Du, Chengkai Sun, Ying Xu, Wensong Ge, Lili Liang, Ruifang Wang, Manqing Sun, Bing Xiao, Wenjuan Qiu

**Affiliations:** 1 Department of Pediatric Endocrinology and Genetic Metabolism, Xinhua Hospital, Shanghai Institute of Pediatric Research, School of Medicine, Shanghai Jiao Tong University, Shanghai, China; 2 Department of Clinical Genetics, Xinhua Hospital, School of Medicine, Shanghai Jiao Tong University, Shanghai, China; 3 Department of Pediatric Internal Medicine, Ruijin Hospital, School of Medicine, Shanghai Jiao Tong University, Shanghai, China; 4 Department of Gastroenterology, Xinhua Hospital, School of Medicine, Shanghai Jiao Tong University, Shanghai, China

## Abstract

**Importance:**

Glycogen storage disease type Ib (GSDIb) is a rare metabolic disorder characterized by impaired glucose-6-phosphate transporter function with limited descriptions.

**Objective:**

To describe the genetic and clinical features of Chinese patients with GSDIb.

**Design, Setting, and Participants:**

This retrospective cohort study retrieved the medical records of 113 Chinese patients with GSDIb treated at a single institution in Shanghai from November 1, 2000, to June 30, 2024.

**Main Outcomes and Measures:**

Biochemical parameters and clinical features (infections, inflammatory bowel disease [IBD], kidney and cardiovascular issues, growth and puberty) at baseline and the last follow-up, all-cause mortality, and mental and social development were assessed.

**Results:**

A total of 113 patients (96 children [85%]; 67 males [59%]) had GSDIb genetically diagnosed at a median age of 1.4 (range, 0.0-35.5) years. Thirty-eight novel *SLC37A4* variants were identified. Hypoglycemia and metabolic derangements were the primary concerns in patients aged 2 years or younger (45%). Short stature (75%), infections (75%), and recurrent epistaxis (31%) became more prevalent in patients aged 2.1 to 5.0 years. The frequency of respiratory tract infection decreased after a median age of 7.0 (IQR, 3.5-10.5) years. Forty-four patients (46%) developed IBD at a median age of 6.0 (IQR, 3.0-12.0) years, 14% of whom developed IBD-associated arthritis at a median age of 10.5 (IQR, 5.8-19.6) years. The mean (SD) difference between the z scores of actual height and target height (Δ height *z* scores) at the final visit (−2.11 [1.76]) did not significantly increase even after uncooked cornstarch treatment. The mean (SD) Δ height *z* scores of the patients with IBD (−2.99 [1.70]) were significantly lower than those without IBD (mean [SD], −1.36 [1.44]) (*P* < .001). Ten patients (9%) died due to complications of GSDIb, including metabolic derangements, sepsis, and/or severe pneumonia (n = 8), IBD (n = 1), and pulmonary hypertension (n = 1).

**Conclusions and Relevance:**

In this cohort study, the genetic and clinical spectra of GSDIb were broadened, suggesting associations between GSDIb and bowel, growth, and survival outcomes. To date, no previous study reported IBD-associated arthritis.

## Key Points

**Question:**

What are the clinical features and genetics of patients with glycogen storage disease type Ib (GSDIb) in China?

**Findings:**

In this cohort study of 113 patients with GSDIb, clinical presentation varied by age, with patients younger than 2 years experiencing mainly hypoglycemia and metabolic derangements; patients 2 to 5 years, infections and epistaxis; patients 5 to 10 years, inflammatory bowel disease; and patients older than 10 years, miscellaneous conditions. In addition, GSDIb was associated with reduced adult height and a 9% mortality rate; 38 novel *SLC37A4* variants were identified.

**Meaning:**

Study results suggest that poor growth, bowel disease, and high mortality may be found in patients with GSDIb.

## Teaser

This cohort study examines genetic factors and clinical characteristics of glycogen storage disease type Ib in Chinese individuals.

## Introduction

Glycogen storage disease type Ib (GSDIb) (OMIM 232220) is an ultrarare inherited disorder affecting glycogenolysis and gluconeogenesis, with an incidence of approximately 1 in 500 000.^1^ It stems from variants in the *SLC37A4* gene, which produces glucose-6-phosphate transporter.^2^ Symptoms of GSDIb include hepatomegaly, nephromegaly, fasting hypoglycemia, lactic acidosis, hyperuricemia, and hyperlipidemia. Recurrent bacterial infections and inflammatory bowel disease (IBD) (Crohn disease–like enterocolitis) due to neutropenia or neutrophil dysfunction can occur.^2,3^ Patients with GSDIb need to maintain normoglycemia through frequent meals, uncooked cornstarch (UCCS), and/or continuous nocturnal gastric drip feeding. In addition, IBD medications and granulocyte colony-stimulating factor (G-CSF) can be administered.^3^ Recently, sodium-glucose cotransporter 2 inhibitors have shown great efficacy and altered the progression of GSDIb.^4^

A study on the clinical course of GSDIb in 57 patients from 16 metabolic centers in 12 European countries was conducted more than 20 years ago^5^; however, to our knowledge, there have been no comparable studies. The present study involved a retrospective analysis of the diagnosis, genotype, management, and clinical course in 113 Chinese patients with GSDIb, with follow-up into adulthood in 17 patients.

## Methods

### Study Design

**Figure 1.**
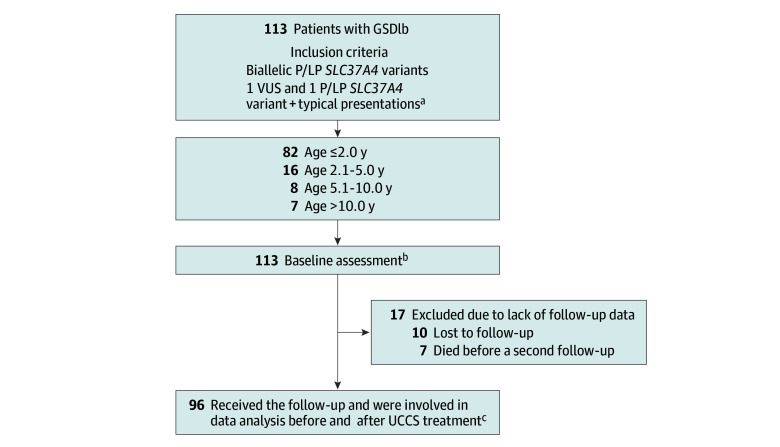
Patient Enrollment and Evaluation GSDIb indicates glycogen storage disease type Ib; P/LP, pathogenic/likely pathogenic; UCCS, uncooked cornstarch; VUS, variant of unknown significance. ^a^Typical presentations including hepatomegaly, elevated liver transaminase levels, hypoglycemia, lactic acidosis, hyperuricemia, and hyperlipidemia. ^b^Baseline assessment data were onset symptoms, laboratory results, and *SLC37A4* variation spectrum. ^c^Data were collected up to the last follow-up before sodium-glucose cotransporter 2 (SGLT2) inhibitor treatment (n = 75) or liver transplant (n = 1), the remaining 20 patients, having neither commenced SGLT2 inhibitor treatment nor undergone liver transplant, were followed up until the end of the study. Components included clinical symptoms and complications, laboratory parameters, and treatment.

This retrospective cohort study described the clinical course of 113 Chinese patients with GSDIb (Figure 1). The Ethical Committee of Xinhua Hospital, Shanghai Jiaotong University School of Medicine, approved this study. Each patient or their legal guardian provided written informed consent. This study followed the Strengthening the Reporting of Observational Studies in Epidemiology (STROBE) reporting guideline.

### Patient Enrollment

A total of 113 patients with GSDIb were identified in the medical records of Xinhua Hospital, Shanghai Jiao Tong University School of Medicine, from November 1, 2000, to June 30, 2024. The inclusion criteria were harboring biallelic pathogenic or likely pathogenic *SLC37A4* variants or presenting clinical symptoms of GSDIb and harboring 1 variant of unknown significance in 1 allele and a pathogenic or likely pathogenic variant in another allele in the *SLC37A4* gene.

The baseline was defined as the clinical data retrieved at diagnosis. Onset symptoms and laboratory results were collected in the baseline assessment. Baseline data were compared among 4 patient groups of different ages: 2.0 years or younger, 2.1 to 5.0 years, 5.1 to 10.0 years, and older than 10.0 years. All patients received UCCS-based dietary treatment after diagnosis following the guideline^2^ (eMethods, eTable 1 in Supplement 1). Other treatments included lipid-lowering agents, urate-lowering agents, G-CSF, and IBD medications.

Among the 113 patients, 17 were lost to follow-up and 96 were evaluated in the follow-up stage, with 76 included until the last follow-up before sodium-glucose cotransporter 2 inhibitor treatment (n = 75) or liver transplant (n = 1), since both interventions would substantially alter the clinical course of GSDIb. The remaining 20 patients were followed up until the end of the study. The assessment indicators and their reference ranges or definitions are listed in eTable 2 in Supplement 1. All-cause mortality and mental and social development were also assessed.

### Molecular Diagnosis

Polymerase chain reaction and Sanger sequencing of the *SLC37A4* gene were performed in 52 patients. Sixty-one patients were tested via exome sequencing (xGen Exome Research Kit; Integrated DNA Technologies), which was performed as previously described.^6,7^ The variants identified by exome sequencing were confirmed by quantitative polymerase chain reaction and Sanger sequencing. The variants were classified following the American College of Medical Genetics and Genomics guidelines.^8^

### Statistical Analysis

Statistical analysis was performed using GraphPad Prism, version 10 (GraphPad Software Inc). Continuous data are expressed as mean (SD) or as median (IQR) and/or range, whereas count data are presented as frequencies and percentages. Paired and unpaired *t* tests were used to compare parametric continuous data at baseline and after treatment, and the Wilcoxon test and the Mann-Whitney test were used to compare nonparametric data. The analysis of variance test was used to assess differences in continuous data between 2 groups. Comparisons of proportions were evaluated via the χ^2^ test. With 2-tailed testing, the level of significance was set at *P* < .05.

## Results

### Demographic Information

The study included 113 Chinese patients with GSDIb from 107 unrelated, nonconsanguineous families (96 children [85%], 17 adults [15%], 67 males [59%], 46 females [41%]). The patients came from 22 provinces, accounting for 65% of the 34 provinces in China. The median age at onset was 0.7 (range, 0.0-35.4) years. Genetic diagnosis was performed at a median age of 1.4 (range, 0.0-35.5) years. Nine of 113 patients (8%) were diagnosed when older than 12 years.

### *SLC37A4* Variation Spectrum

**Figure 2.**
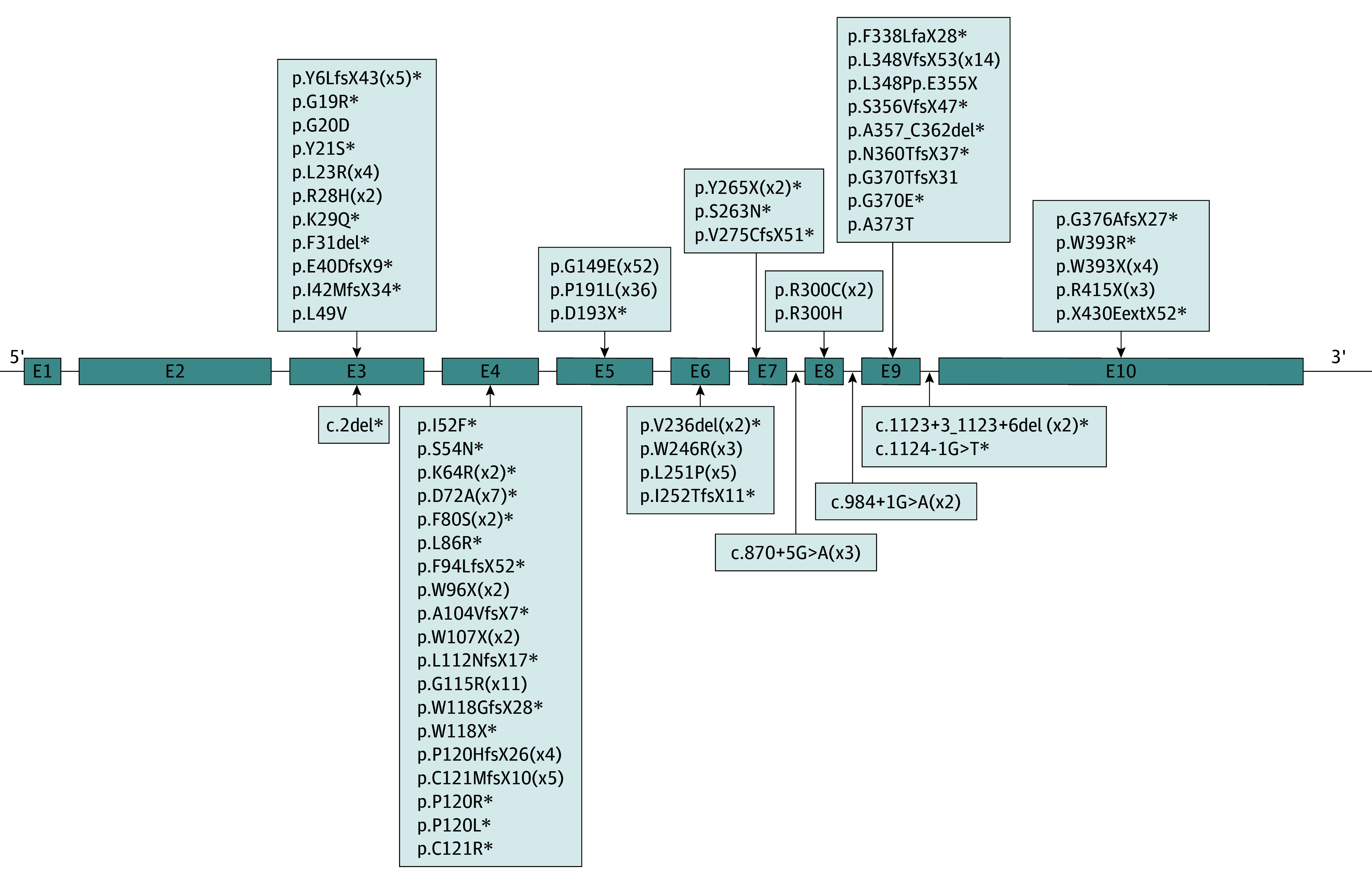
Variants in the *SLC37A4* Gene Identified in 113 Patients With Glycogen Storage Disease Type Ib (GSDIb) The *SLC37A4* gene is exhibited as a line diagram with the 10 exons (E) marked as gray boxes. Variants are depicted as nucleotide alterations and amino acid alterations per exon/intron. Novel variants are marked with an asterisk. Suffixes indicate the number of mutant alleles.

In patients with GSDI confirmed by genetic diagnosis at our center, GSDIb accounted for 18% (113 of 616). Sixty-two *SLC37A4* variants identified in these patients included 28 missense, 17 frameshift, 8 nonsense, 4 splicing site, 3 in-frame, 1 stop retained, and 1 initiator codon variant, including 38 novel variants (NM_001164277.2) (Figure 2; eTable 3 in Supplement 1).

The hotspot variants recognized were p.G149E (52 of 214 [24%]) and p.P191L (36 of 214 [17%]); p.G149E primarily originated from Southern China (45 of 52 [87%]), especially Guangdong. Among the parents of patients, 54 were from Guangdong, and 38 of them (70%) carried p.G149E, which showed a possible founder effect in Guangdong; p.P191L was spread in 16 provinces (eFigure 1 in Supplement 1). The gnomAD revealed a higher frequency of p.G149E (4.043 × 10^−4^ vs 2.921 × 10^−5^) and p.P191L (2.231 × 10^−5^ vs 6.198 × 10^−7^) in East Asians than in all races.

### Clinical Features at Baseline

At baseline, the dominant presentations included abdominal protrusion (95%), hypoglycemia and/or metabolic derangements (37%), infections (55%), short stature (51%), diarrhea (42%), and recurrent epistaxis (11%) (Table). Other initial presentations included neutropenia (n = 3, all 0.6 years old), fever after vaccination (n = 2, 0.4-0.6 years old), IBD-related abdominal pain (n = 2, 8.0-12.7 years old), liver adenomas (n = 1, 12.0 years old), gout (n = 1, 22.0 years old), and an incident finding by prenatal genetic diagnosis due to abnormal ultrasonography results (n = 2, monozygotic twins).

**Table.** Clinical Information at Baseline and After UCCS Treatment of 113 Patients With GSDIb

Presentation	At baseline, percentage, No./total No. (%)					After UCCS treatment, overall (n = 96)	*P* value	
	≤2.0 y (n = 82)	2.1-5.0 y (n = 16)	5.1-10.0 y (n = 8)	>10.0 y (n = 7)	Overall (n = 113)		Baseline vs treatment	Intergroup comparison^a^
Abdominal protrusion	76/82 (93)	16/16 (100)	8/8 (100)	7/7 (100)	107/113 (95)	96/96 (100)	.03	.49
Hypoglycemia/metabolic derangements	37/82 (45)	3/16 (19)	1/8 (13)	1/7 (14)	42/113 (37)	45/96 (47)	.16	.04
Infections	41/82 (50)	12/16 (75)	7/8 (88)	2/7 (29)	62/113 (55)	77/96 (80)	<.001	.03
Short stature	33/82 (40)	12/16 (75)	7/8 (88)	6/7 (86)	58/113 (51)	50/96 (52)	>.99	.002
Recurrent epistaxis	3/82 (4)	5/16 (31)	2/8 (25)	2/7 (29)	12/113 (11)	2/96 (2)	.02	.001
Diarrhea	32/82 (39)	7/16 (44)	5/8 (63)	3/7 (43)	47/113 (42)	59/96 (62)	.005	.64

Abbreviations: GSDIb, glycogen storage disease type Ib; UCCS, uncooked cornstarch.

^a^ Examining variations in presentation prevalence across 4 age groups at baseline.

Patients were divided into 4 groups based on age at baseline: 2.0 years or younger (n = 82), 2.1 to 5.0 years (n = 16), 5.1 to 10.0 years (n = 8), and older than 10.0 years (n = 7). There were significant differences in the ratios of short stature, infections, and recurrent epistaxis among the different groups (Table). Short stature (≤2 years or younger: 40% vs 2.1-5.0 years, 75%; *P* = .01; 40% vs 5.1-10.0 years, 88%; *P* = .02; 40% vs >10.0 years, 86%; *P* = .04) and recurrent epistaxis (≤2 years, 4% vs 2.1-5.0 years, 31%; *P* = .003; 4% vs >10.0 years, 29%; *P* = .048) were more common in patients older than 2 years old, while the prevalence of infections significantly decreased in patients older than 10 years (5.1-10.0 years: 88% vs >10.0 years: 29%; *P* = .04). These patients presented differently depending on their age: hypoglycemia and metabolic derangements were the primary concerns in the 2.0 years or younger group (45%); short stature (75%), recurrent epistaxis (31%), and infections (75%) became more prevalent in the age 2.1 to 5.0 years group; IBD flared in the age 5.1 to 10.0 years group, along with the prevalence of short stature increasing. The presentations in patients older than 10 years were miscellaneous, including abdominal protrusion, IBD, gout, and liver adenomas.

All patients experienced fasting hypoglycemia. Neonatal hypoglycemia was observed in 13% (15 of 113) of the patients, neutropenia was reported in 78%, and anemia was reported in 67%. The median alanine aminotransferase (ALT), aspartate aminotransferase (AST), γ-glutamyl transpeptidase (GGT), lactate, uric acid *z* score, total cholesterol, and triglyceride levels increased (eTable 4 in Supplement 1). The ALT, AST, and GGT levels at baseline tended to decrease with age (eFigure 2 in Supplement 1). These data were significantly higher in patients aged 2 years or younger than in those older than 10 years (median ALT: 107.0 vs 54.5 U/L; *P* = .02; AST: 178.5 vs 64.0 U/L; *P* = .002; GGT: 220.0 vs 69.5 U/L; *P* = .001 [for all values, to convert to per microkatals per liter, multiply by 0.0167]). All patients had hepatomegaly on presentation.

### Clinical Features at the Follow-Up Assessment

#### Dietary Treatment and Biochemical Control

Ninety-six patients were followed up to a median age of 9.1 (IQR, 3.3-15.5) years, totally for a median of 8.4 (IQR, 4.9-13.1) years. All patients received UCCS treatment at a median dose of 2.0 g/kg (IQR, 1.6-2.0 g/kg) 3 to 6 times per day. Two patients were fed continuous nocturnal gastric drip feeding at night due to severe IBD. Of 96 patients, 68 (71%) used a continuous real-time blood glucose monitoring system (24% [23 of 96]) or invasive blood glucose meter (47% [45 of 96]). In the preceding 6 months, hypoglycemia occurred 1 to 2 (64% [29 of 45]), 3 to 4 (22% [10 of 45]), or more than 5 times (13% [6 of 45]). Causes of hypoglycemia included delayed nocturnal feeding (62% [28 of 45]), increased exercise (7% [3 of 45]), episodes of IBD (16% [7 of 45]), infections (11% [5 of 45]), urolithiasis (2% [1 of 45]), and hernia (2% [1 of 45]) (eTable 1 in Supplement 2).

At the last follow-up, ALT, AST, GGT, lactate, uric acid *z* score, total cholesterol, and triglyceride levels all significantly decreased (eFigure 2, eTable 4 in Supplement 1). Gout was present in 12% (11 of 96) of patients at a median age of 17.8 (IQR, 9.3-19.0) years. Hepatic adenomas were discovered in 10% (10 of 96) of the patients at a mean (SD) age of 20.7 (9.7) years.

#### Neutropenia and Infections

**Figure 3.**
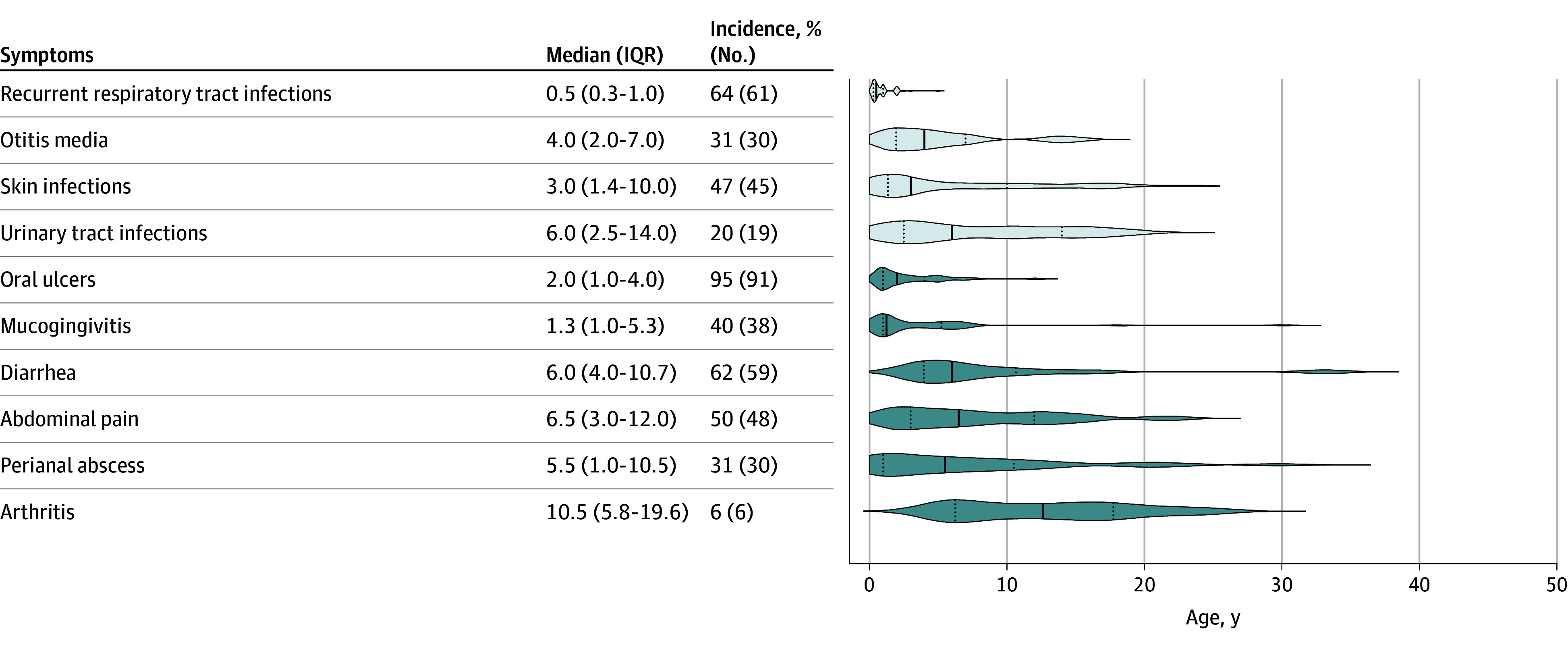
Age at Onset of Infections and Inflammatory Bowel Disease (IBD)-Related Symptoms of Patients With Glycogen Storage Disease Type Ib Infections (light gray violins) and IBD-related symptoms (dark gray violins) in 96 patients. Medians are indicated as solid lines and quartiles as dashed lines within the violins.

At the final follow-up, neutropenia was detected in 77% (74 of 96) of patients and severe neutropenia in 32% (31 of 96). Infections included recurrent respiratory tract infections (RTIs) (64% [61 of 96]), otitis media (31% [30 of 96]), skin infections (47% [45 of 96]) (eTable 2 in Supplement 2), and urinary tract infections (20% [19 of 96]) (Figure 3). Recurrent RTIs were present at a median age of 0.5 (IQR, 0.3-1.0) years in 64% (61 of 96) of the patients. Among them, 61% (37 of 61) exhibited a decrease of RTI frequency and no longer satisfied the diagnostic criteria for recurrent RTIs after a median age of 7.0 (IQR, 3.5-10.5) years. By the end of follow-up, the prevalence of recurrent RTIs significantly decreased to 25% (24 of 96; *P* < .001).

Among the 74 patients with neutropenia, only 18 (24%) used G-CSF due to cost and concerns about the adverse effects. The mean (SD) duration of G-CSF treatment was 5.1 (4.4) years (eTable 3 in Supplement 2). Only 6 patients experienced a reduction in RTIs after G-CSF treatment, suggesting the decrease in RTIs in patients with GSDIb might be attributed to some age-related factors. Splenomegaly was detected in 33% (6 of 18) of the patients who received G-CSF treatment via ultrasonography.

#### Inflammatory Bowel Disease

Crohn disease–like IBD was diagnosed in 44 of 96 patients (46%) at a median age of 6.0 (IQR, 3.0-12.0) years, including 33 pediatric and 11 adult patients (eTable 4 in Supplement 2). Twenty-three (44%) of the 52 patients without IBD were younger than 6 years. At the time of the initial IBD assessment, the median Pediatric Crohn’s Disease Activity Index (PCDAI) of pediatric patients was 60.0 (IQR, 47.5-70.0) (severe ≥40, moderate 30-37.5, mild 10-27.5, remission <10^9^). The mean (SD) Crohn’s Disease Activity Index (CDAI) of adult patients was 370.4 (113.1) (severe >450, moderate 221-450, mild 150-220, remission <150^10^). Thirty-one patients had severe IBD, 8 had moderate IBD, and 5 had mild IBD. Fecal calprotectin levels were increased in 77% (10 of 13) of the patients. Three patients underwent intestinal resection and enterostomy for intestinal obstruction at ages 13 to 23 years. Endoscopy was performed in 28 patients and revealed ulcers and congestion, edema, erosion, blurred vascular markings of the colonic mucosa (n = 28), cobble-stoning of the mucosa (n = 2), and stenosis of the ileocecal valve (n = 1) and ascending (n = 1)/transverse (n = 1) colon. Data concerning the IBD-related symptoms of the 96 patients are provided in Figure 3. Oral ulcers, the most common IBD-related symptom, occurred in 95% (91 of 96) of the patients at age 2.0 (IQR, 1.0-4.0) years and developed in all patients with IBD.

Twenty-four of 44 patients (55%) with IBD received drugs including G-CSF (n = 11), mesalazine (n = 5), G-CSF and mesalazine (n = 4), thalidomide (n = 2), adalimumab (n = 1), and G-CSF and adalimumab (n = 1) (eTable 5 in Supplement 2). The remaining 20 patients refused IBD treatment. Data concerning IBD treatment for 13 patients were missing. The mean (SD) PCDAIs significantly decreased from 69.6 (8.9) to 48.2 (13.0) in 7 pediatric patients treated with mesalazine (*P* = .007). The mean (SD) PCDAIs of 6 pediatric patients considerably decreased from 67.0 (9.9) to 51.0 (5.2) after G-CSF treatment (*P* = .003). These findings suggest that mesalazine and G-CSF could partially improve IBD in patients with GSDIb.

Inflammatory bowel disease–associated arthritis occurred in 14% (6 of 44) of the patients at a median age of 10.5 (IQR, 5.8-19.6) years, with a median time of 3.5 (IQR, 1.5-6.9) years after their IBD diagnosis. The episode of arthritis was persistent (lasting >4 months, n = 3) or lasted for 1 (n = 2) to 4 months (n = 1) (eTable 5 in Supplement 1). Rheumatoid factor, human leukocyte antigen-B27, and autoantibodies were all negative. Two patients received adalimumab and/or G-CSF for persistent arthritis. The CDAIs of these patients decreased, and arthritis greatly improved after treatment.

#### Kidney Issues

Microalbuminuria was detected in 46% of the patients (23 of 50) at a mean (SD) age of 11.8 (6.8) years, and proteinuria was detected in 48% (24 of 50) at a mean (SD) age of 14.0 (9.9) years. A decrease in the estimated glomerular filtration rate was not observed. Urolithiasis developed in 14% (13 of 96) of the patients at a mean (SD) age of 17.6 (7.0) years.

#### Cardiovascular Issues

One patient presented with pulmonary hypertension at age 25 years. Bosentan and sildenafil were administered, but he died from heart failure 6 months after the diagnosis of pulmonary hypertension during the SARS-CoV-2 pandemic. Another patient developed hypertension at age 36 years.

#### Growth and Puberty

**Figure 4.**
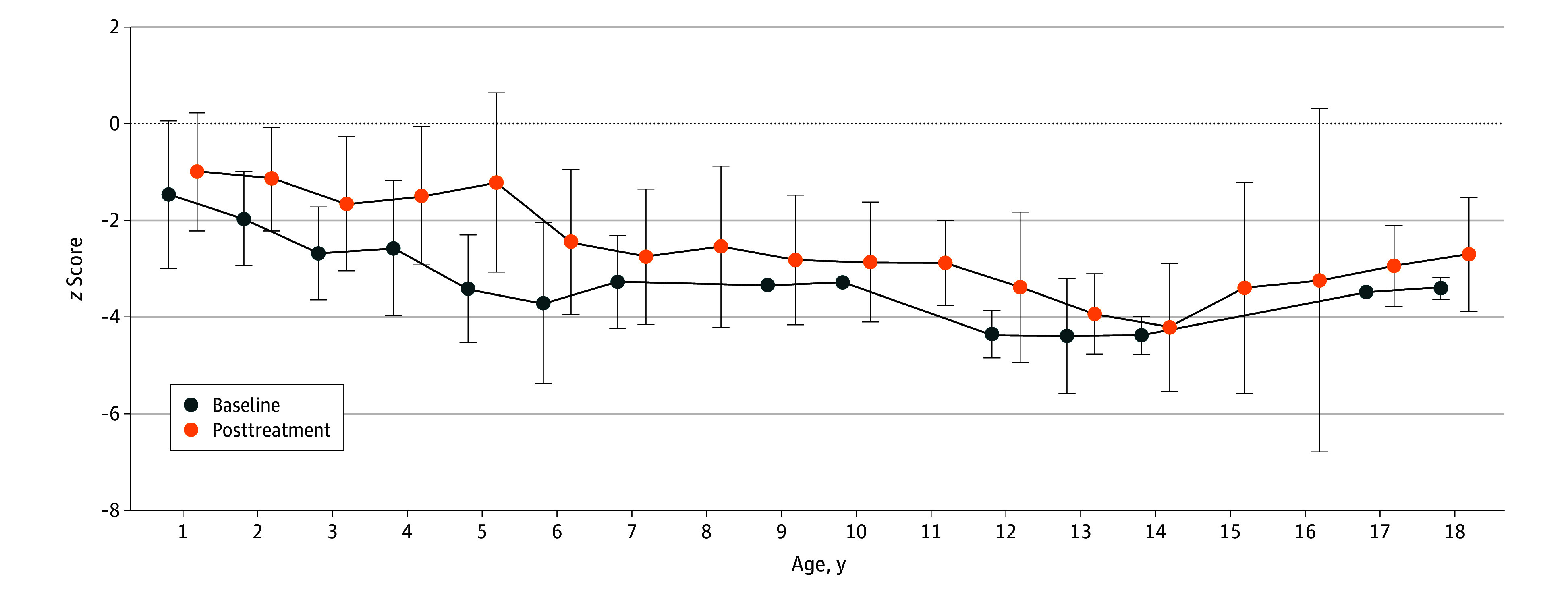
Δ Height *z* Scores vs Age in Patients With Glycogen Storage Disease Type Ib Mean Δ height *z* scores per-year interval at baseline and after treatment. Error bars indicate SD.

Height measurements of patients with GSDIb at baseline and during the follow-up were categorized per-year interval and are presented as the mean (SD) Δ height *z* scores (the difference between the z scores of actual height and target height, calculated as [mother's height + father's height]/2 ± 6.5 cm) (Figure 4; eTable 6 in Supplement 1). Three patients had already reached adult height at baseline and thus were not included in the comparison of Δ height. Patients aged 2, 3, and 5 years after UCCS treatment had significantly higher Δ height *z* scores than those before UCCS treatment, indicating the association between hypoglycemia and UCCS treatment with growth. The median age at IBD onset in this cohort was 6 years. The mean (SD) Δ height *z* scores of 43 patients with IBD (−2.99 [1.70]) were significantly lower than those of 50 patients without IBD (−1.36 [1.44]) at the final visit (*P* < .001), indicating the association between IBD and growth. At the last follow-up, there were no significant changes in the Δ height *z* score (mean [SD], baseline: n = 99; −2.22 [1.40]; final visit: n = 93; −2.11 [1.76]; *P* = .64) and body mass index *z* score (mean [SD], baseline: n = 101; 0.22 [1.41]; final visit: n = 96; 0.34 [1.51]; *P* = .55), indicating the association between GSDIb and growth. Nineteen patients reached adult height (mean [SD], −2.32 [1.24]), and 12 had short stature.

Osteopenia (mean [SD] bone mineral density *z* score: −3.37 [0.90]) was identified in 56% (10 of 18) of the patients at a mean (SD) age of 18.6 (6.2) years. Insulinlike growth factor-1 (IGF-1) levels in 6 of 12 patients were lower than those of healthy counterparts. Thirty-one patients entered puberty at a mean (SD) age of 14.6 (2.3) years (n = 20) for males and 11.4 (1.8) years (n = 11) for females. Pubertal development was delayed in 45% of males (9 of 20) and 18% of females (2 of 11).

#### Liver Transplant

One patient underwent a liver transplant at age 4 years 4 years before June 2024. No liver transplant–related complications were reported. Following the transplant, UCCS and G-CSF treatments were discontinued. At the final visit, the RTI frequency decreased, and the Δ height *z* score increased. The absolute neutrophil count increased but remained below the reference range. The PCDAI level decreased but did not reach the level of IBD remission (eTable 7 in Supplement 1).

#### All-Cause Mortality

In total, 11 of the 113 patients with GSDIb died (10%). Except for 1 death resulting from a car crash, the remaining 10 deaths (9%) were attributed to GSDIb-related complications: metabolic derangements, sepsis/severe pneumonia (n = 8), IBD (n = 1), and pulmonary hypertension (n = 1). Four patients died when they were younger than 1 year, 2 aged 1 to 3 years, 2 aged 7 years, and 2 older than 20 years. Nine of the 10 patients had not received G-CSF or IBD treatment (eTable 8 in Supplement 1).

#### Mental and Social Development

Most patients had normal mental development. Five school-aged patients (4%) could not receive normal general education due to IBD (with G-CSF/IBD treatment, n = 2; without G-CSF/IBD treatment, n = 1), recurrent infections (without G-CSF treatment, n = 1), and low intelligence (n = 1, Wechsler Intelligence Scale for Children score: 50 at age 13 years) (eTable 9 in Supplement 1). Among 17 adult patients, 8 were undergraduates, 4 were employed, and 5 were unemployed.

## Discussion

This study included 113 Chinese patients with GSDIb from 22 provinces and retrospectively examined their infections, IBD, biochemical control, growth, and mortality over a 24-year follow-up period. Ten patients (9%) died from complications of GSDIb, comparable to an earlier study (9% [5 of 57]).^5^ Among them, 4 died within age 1 year from metabolic derangements, sepsis, and/or pneumonia. Of these 4 patients, GSDIb was undiagnosed in 3 before their deaths. Thus, the mortality of GSDIb might be even greater. Timely diagnosis and intervention are important for decreasing the mortality of GSDIb. Furthermore, 4 deceased siblings of 4 patients who lacked complete medical records and genetic diagnosis are believed to have died due to metabolic derangements caused by GSDIb. This suspicion is based on their characteristic GSDI symptoms, including lactic acidosis and hypoglycemia, as well as the fact that the siblings all have a genetic diagnosis of GSDIb, which is an autosomal recessive condition. In this cohort, neonatal hypoglycemia was detected in 13% of the patients, but none of them received a diagnosis during the neonatal period. The diagnosis was delayed by at least 2 months, or even 9.6 years, and established after patients experienced other symptoms, such as abdominal protrusion and metabolic derangements. Therefore, neonatal hypoglycemia could be an indicator of GSDI and requires timely clinical examinations and etiologic identification. Additionally, our results showed liver enzyme levels might decrease with age. This trend could also be associated with a milder clinical phenotype in some older individuals, potentially leading to a later diagnosis.

Our study provides a review of RTI frequency and onset age in patients with GSDIb. The RTI frequency in these patients gradually decreased after they were aged 7 years, and this pattern did not align with G-CSF administration. These results suggest the RTI frequency of patients with GSDIb may naturally decrease with age, often occurring after the start of the school-age period, although these patients still presented with neutropenia. Previous studies have reported some older patients with GSDIb do not experience frequent RTIs,^11^ presumably because the immune system matures and other immune components can defend against infections. For example, age is positively correlated with C3 levels,^12^ a key component in the complement system that lyses bacteria.

In this study, IBD developed in 46% of the patients when they were approximately aged 6 years. This incidence is higher than previous reports (24%-35%) at age 1.8 to 7.0 years.^11,13,14,15,16,17,18^ Furthermore, 44% of our non-IBD patients were younger than 6 years. We speculate that nearly half of these patients will develop IBD in the future without specific treatment. Oral ulcers, the most common IBD-related symptom, occurred in 95% of patients at approximately age 2 years and developed in all patients with IBD. In contrast, the incidence of oral ulcers in classical Crohn disease is only 8% to 48%, and the onset age is approximately 12 years,^19,20,21^ revealing the distinctiveness of IBD in patients with GSDIb. Furthermore, to our knowledge, this study is the first to report IBD-associated arthritis. Among the 6 patients with arthritis, only 2 had received G-CSF treatment, while the remaining 4 had not undergone any IBD treatment before the onset of arthritis. Therefore, the arthritis in these patients may be reactive arthritis caused by untreated or severe IBD.

An association between GSDIb and growth was observed (mean [SD] Δ height *z* score: −2.11 [1.76]). Treatment with UCCS could temporarily increase height in younger patients at ages 2, 3, and 5 years, but it did not significantly increase the height at all ages and the adult height of GSDIb (−2.32). These findings suggested their height was influenced by multiple factors other than hypoglycemia, especially IBD. This was supported by the finding that the IBD patients in our cohort (−2.99) were significantly shorter than those without IBD (−1.36) at the final visit. Prior research also reported that patients with GSDIb (−2.08) were shorter than those with GSDIa (−1.44) without IBD.^2^ Overall, hypoglycemia, IBD, decreased insulinlike growth factor–1,^22^ osteopenia, and delayed puberty^2^ may be attributed to the short stature of patients with GSDIb.

### Limitations

Certain limitations of our study merit attention. Many medical records were incomplete in this study. The inclusion of baseline data from 17 patients lost to follow-up may introduce biases. Only 24% of patients had access to G-CSF therapy, and 55% received IBD therapy, which was far below the standard for these conditions. Additionally, 8% had delayed diagnosis (diagnosed when aged >12 years). Compared with cohorts from other countries, the cohort in the present study had a higher proportion of IBD and what appears to be a newly recognized complication—arthritis. These more severe phenotypes may be related to the lower proportion of standard G-CSF and IBD treatments among these patients. Furthermore, most of the cohort were preadolescent, underscoring the need for ongoing follow-up.

## Conclusions

This retrospective analysis of 113 patients with GSDIb depicted their presentations and management, broadening the genetic and clinical spectra of GSDIb. Respiratory tract infections occurred in 64% of patients at age 0.5 years, and might naturally decrease by age 7 years, IBD developed in 46% at approximately age 6 years, and IBD-associated arthritis, occurred in 14% at age 10.5 years old. These findings suggest that GSDIb may be associated with growth and survival.

## Notes

Supplement 1. **eMethods.** Dietary Treatment and Follow-Up **eTable 1.** Inpatient Management of GSDI Acute Metabolic Derangement **eTable 2.** Assessment Indicators of GSDIb Patients **eTable 3.** *SLC37A4* Variants in 113 GSDIb Patients **eTable 4.** Laboratory Data at Baseline and After UCCS Treatment of 113 GSDIb Patients **eTable 5.** Details of Arthritis in Six GSDIb Patients **eTable 6.** Mean ΔHeight *z* Scores per-Year Interval at Baseline and After UCCS Treatment in GSDIb Patients **eTable 7.** Clinical Changes in P56 Before and After Liver Transplant **eTable 8.** Details of Deceased GSDIb Patients **eTable 9.** Details of Five Dropout GSDIb Patients **eFigure 1**. Geographical Distribution of Patients’ Origins, p.G149E and p.P191L in China **eFigure 2.** Biochemical Control of GSDIb Cohort
eReferences

Supplement 2. **eTable 1.** Blood Glucose Monitoring and Hypoglycemia Incidence in 96 GSDIb Patients **eTable 2.** History of Skin Infection in 45 GSDIb Patients **eTable 3.** Neutropenia Incidence and G-CSF Treatment in 96 GSDIb Patients **eTable 4.** IBD-Related Symptoms and Indicators in 44 GSDIb Patients With IBD **eTable 5.** Inflammatory Indicators and Treatments in 44 GSDIb Patients With IBD

Supplement 3.
Data Sharing Statement
