# Supplementary material for: Genetic Variants and Clinical Features of Patients With Glycogen Storage Disease Type Ib
Source: JAMA Netw Open. 2025 Feb 26;8(2):e2461888. doi: 10.1001/jamanetworkopen.2024.61888 (PMC11866026; doi:10.1001/jamanetworkopen.2024.61888)
Supplement: Supplement 1. — eMethods. Dietary Treatment and Follow-Up eTable 1. Inpatient Management of GSDI Acute Metabolic Derangement eTable 2. Assessment Indicators of GSDIb Patients eTable 3. SLC37A4 Variants in 113 GSDIb Patients eTable 4. Laboratory Data at Baseline and After UCCS Treatment of 113 GSDIb Patients eTable 5. Details of Arthritis in Six GSDIb Patients eTable 6. Mean ΔHeight z Scores per-Year Interval at Baseline and After UCCS Treatment in GSDIb Patients eTable 7. Clinical Changes in P56 Before and After Liver Transplant eTable 8. Details of Deceased GSDIb Patients eTable 9. Details of Five Dropout GSDIb Patients eFigure 1. Geographical Distribution of Patients’ Origins, p.G149E and p.P191L in China eFigure 2. Biochemical Control of GSDIb Cohort eReferences [file jamanetwopen-e2461888-s001.pdf]

Supplementary Online Content

Xia Y, Sun Y, Du T, et al. Genetic variants and clinical features of patients with glycogen storage disease type Ib. *JAMA Netw Open*. 2025;8(2):e2461888. doi:10.1001/jamanetworkopen.2024.61888

eMethods. Dietary Treatment and Follow-Up
eTable 1. Inpatient Management of GSDI Acute Metabolic Derangement

eTable 2. Assessment Indicators of GSDIb Patients

eTable 3. *SLC37A4* Variants in 113 GSDIb Patients

eTable 4. Laboratory Data at Baseline and After UCCS Treatment of 113 GSDIb Patients

eTable 5. Details of Arthritis in Six GSDIb Patients

eTable 6. Mean ΔHeight *z* Scores per-Year Interval at Baseline and After UCCS Treatment in GSDIb Patients

eTable 7. Clinical Changes in P56 Before and After Liver Transplant

eTable 8. Details of Deceased GSDIb Patients

eTable 9. Details of Five Dropout GSDIb Patients

eFigure 1. Geographical Distribution of Patients' Origins, p.G149E and p.P191L in China

eFigure 2. Biochemical Control of GSDIb Cohort

eReferences

This supplementary material has been provided by the authors to give readers additional information about their work.

© 2025 Xia Y et al. *JAMA Network Open.*

eMethods. Dietary Treatment and Follow-up

Dietary treatment is given once the patient was clinically diagnosed with glycogen storage disease type I (GSDI). Based on the GSDI guideline of American College of Medical Genetics and Genomics (ACMG) [1], our department recommended a diet consisting of small frequent feedings, as follows: carbohydrates account for 60-70% of the dietary calories, protein for 10-15%, and fat for the remaining portion (<30% for pediatric patients over two years). Infant patients are fed every 2-3 hours with formulas that are free of sucrose, fructose, and lactose. Uncooked cornstarch (UCCS) is gradually introduced to patients over six months old and consumed every 3-5 hours. Foods that contains sucrose, fructose and lactose are eliminated from the diet. Vitamin D is supplemented. Blood glucose is monitored by invasive blood glucose meter or continuous real-time blood glucose monitoring system. The recommended preprandial glucose level is 4-6 mmol/L. A standard inpatient protocol is designed and provided for hospitalization for metabolic derangements (eTable 1).

© 2025 Xia Y et al. *JAMA Network Open.*

eTable 1. Inpatient Management of GSDI Acute Metabolic Derangement

Condition	Detailed Description	
Hypoglycemia	Diagnosis standard	Blood glucose < 59.5 mg/dL (3.3 mmol/L)[2]
	Symptoms	Lethargy, fatigue, nausea, irritability, dizziness, sweating
	Measures	Provide 50% glucose solutions (10mL) to rapidly increase blood glucose, then maintain blood glucose through snacks or UCCS
		If unable to tolerate enteral nutrition, administer intravenous injection of 10% glucose solutions (2-4mL/kg) and subsequent continuous infusion of 10-12.5% glucose (GIR: 5-8mg/kg/min) until enteral nutrition is tolerated again
Lactic acidosis	Diagnosis standard	pH ≤ 7.35 + blood lactate level > 18.02 mg/dL (2 mmol/L) + PaCO2 ≤ 42 mmHg[3]
	Symptoms	Dyspnea, vomiting, anorexia, lethargy
	Measures	Administer intravenous infusion of 10-12.5% glucose solutions with appropriate electrolytes (GIR: neonate 10mg/kg/min, infant 8mg/kg/min, > one year old 6mg/kg/min)
		If blood glucose > 144.1 mg/dL (8 mmol/L), provide insulin (0.05-0.1U/kg/h) and potassium
		If pH < 7.30, provide sodium bicarbonate
		Provide blood purification treatment if blood lactate level does not decrease less than 30% or remains over 135.1-180.2 mg/dL (15-20 mmol/L)
		Energy intake (enteral + parenteral): 60-100 kcal/kg/d
		Provide oxygen therapy or mechanical ventilation when dyspnea occurs

GSDI: glycogen storage disease type I, GIR: glucose infusion rate, PaCO2: partial pressure of carbon dioxide, UCCS: uncooked cornstarch.

© 2025 Xia Y et al. *JAMA Network Open.*

eTable 2 Assessment Indicators of GSDIb Patients

	Indicator	Reference range/definition
Clinical evaluation	Onset symptoms	NA
	History of infections	Recurrent RTIs[4]: upper RTIs > 7 episodes/year at 0-2 years old<br>> 6 at 2-5 years old<br>> 5 at 5-14 years old<br>and/or lower RTIs > 2 episodes per year
		Otitis media
		Urinary tract infections
		Skin infections: meeting at least one of the following criteria<br>i. The frequency of infection was greater than or equal to three times/year[5];<br>ii. The infection persisted for more than 10 days and needed wound disinfection and antibiotic ointment treatment;<br>iii. The infection required oral/intravenous antibiotics or surgical treatment.
	History of IBD-related symptoms[#]	Abdominal pain
		Diarrhea[6]: ≥ 3 loose or watery stools a day lasting for > 2 weeks
		Perioral infections: oral ulcers, mucogingivitis
		Perianal lesions: abscesses, fistulas, fissures
		Weight loss
	Recurrent epistaxis	≥5 times per year[7]
	Δ Height*^	Short stature: z score ≤-2[8]
	BMI*	z score -2 ~ 2[8,9]
	Prevalence of neonatal hypoglycemia	NA
	Records of prescription utilization	NA
	Records of blood glucose monitoring and metabolic derangements	NA
Laboratory tests	Blood glucose	≥ 59.5 mg/dL (3.3 mmol/L)[2]
	Blood lactate	< 18.02 mg/dL (2.0 mmol/L)[3]
	ALT	< 75 U/L
	AST	< 35 U/L

© 2025 Xia Y et al. *JAMA Network Open.*

eTable 2 Assessment Indicators of GSDIb Patients (continued)

	Indicator	Reference range/definition
Laboratory tests	GGT	< 73 U/L
	TC	< 239.38 mg/dL (6.2 mmol/L) [10]
	TG	< 204.42 mg/dL (2.31 mmol/L) [10]
	UA*	z score -2 ~ 2[11]
	Absolute neutrophil count	Neutropenia: < 1500.0 μL (1.5 × 10^9/L)<br>Severe neutropenia: < 500.0 μL (0.5 × 10^9/L) [12]
	Hemoglobin	Anemia: hemoglobin < 11.0 g/dL[13]
	C-reactive protein	< 1.0 mg/dL
	Erythrocyte sedimentation rate	< 20 mm/h
	IGF-1[14]	NA
	Urinary Albumin/creatinine ratio	Microalbuminuria: 2.5-20 mg/g<br>Proteinuria: >20 mg/g[15]
	Fecal calprotectin	< 50 ug/g
	Estimated glomerular filtration rate**	≥ 90 ml/min/1.73 m^2 [16]
	Ultrasonography	Evaluation of hepatomegaly, liver adenomas, splenomegaly, perianal abscess, and urolithiasis
	Gastrointestinal endoscopy with biopsies	NA
	Contrast-enhanced CT for the small intestine	NA
	Bone mineral density*	Osteopenia: z score ≤ -2[17]

RTIs: respiratory tract infections; ALT: alanine aminotransferase; AST: aspartate aminotransferase; GGT: γ-glutamyl transpeptidase; TC: total cholesterol; TG: triglycerides; UA: uric acid; IBD: inflammatory bowel disease; IGF-1: insulin-like growth factor-1; BMI: body mass index, calculated as weight (in kg)/ $height^2$ (in m^2); CT: computed tomography; NA: not applicable.

*: transformed into z score according to the corresponding reference values for different age and sex groups.

**: estimated glomerular filtration rate was calculated the Chronic Kidney Disease Epidemiology Collaboration equation[18] in adults and the Schwartz equation[19] in children.

^: Height z score was computed for each 1-year interval. The z score of Δheight was defined as the difference between the z scores of actual height and target height (calculated as [mother's height

© 2025 Xia Y et al. *JAMA Network Open.*

+ father's height]/2 ± 6.5 [in cm]).
#: IBD is considered when one or more IBD-related symptoms last for over four weeks or occurs for more than twice within six months, associated with laboratory assessment (erythrocyte sedimentation rate, fecal calprotectin, gastrointestinal endoscopy with biopsies, and contrast-enhanced CT for the small intestine) [20]. Disease activity was assessed using the weighted Pediatric Crohn's Disease Activity Index (PCDAI) [21] (severe ≥40, moderate 30-37.5, mild 10-27.5, remission < 10) for patients aged under 18 years and the Crohn's Disease Activity Index (CDAI) (severe >450, moderate 221-450, mild 150-220, remission <150) for those aged over 18 years[22] based on the IBD phenotyping guideline[23].

© 2025 Xia Y et al. *JAMA Network Open.*

eTable 3 *SLC37A4* Variants in 113 GSDIb Patients

Patient ID	Paternal origin	Maternal origin	Allele 1 (paternal)				Allele 2 (maternal)			
			Nucleotide alteration (NM_001164277.2)	Amino acid alteration	Classification	ref.	Nucleotide alteration (NM_001164277.2)	Amino acid alteration	Classification	ref.
P1	Henan	Henan	c.1042_1043del	p.L348VfsX53	P	[24]	c.1287_1290del	p.X430EextX52	P	this study
P2	Guangxi	Guangxi	c.446G>A	p.G149E	P	[25]	c.795C>G	p.Y265X	P	this study
P3	Anhui	Anhui	c.215A>C	p.D72A	P	this study	c.68T>G	p.L23R	LP	[26]
P4	Henan	Henan	c.572C>T	p.P191L	P	[27]	c.752T>C	p.L251P	P	LOVD (#0000874563)
P5	Henan	Henan	c.898C>T	p.R300C	LP	[26]	c.1117G>A	p.A373T	VUS	ClinVar
P6	Henan	Henan	c.215A>C	p.D72A	P	this study	c.359del	p.P120HfsX26	P	[32]
P7	Jiangsu	Jiangsu	c.572C>T	p.P191L	P	[28]	c.1042_1043del	p.L348VfsX53	P	[29]
P8	Jiangxi	Guangdong	c.359dupC	p.C121MfsX10	P	[30]	c.446G>A	p.G149E	P	[31]
P9	Shaanxi	Shaanxi	c.572C>T	p.P191L	P	[27]	c.572C>T	p.P191L	P	[27]
P10	Shandong	Shandong	c.572C>T	p.P191L	P	[27]	c.1042_1043del	p.L348VfsX53	P	[24]
P11	Hunan	Hunan	c.446G>A	p.G149E	P	[31]	c.1124-1G>T	/	P	this study
P12	Guangxi	Guangdong	c.446G>A	p.G149E	P	[31]	c.446G>A	p.G149E	P	[31]
P13	Hunan	Hunan	c.1042_1043del	p.L348VfsX53	P	[24]	c.706_708del	p.V236del	LP	this study
P14	Heilongjiang	Heilongjiang	c.359del	p.P120HfsX26	P	[32]	c.572C>T	p.P191L	P	[27]
P15	Guizhou	Guizhou	c.572C>T	p.P191L	P	[28]	c.870+5G>A	/	VUS	[26]
P16	Sichuan	Sichuan	c.333_344delinsGAACTGG	p.L112NfsX17	P	this study	c.282del	p.F94LfsX52	P	this study
P17	Guangdong	Guangdong	c.446G>A	p.G149E	P	[31]	c.446G>A	p.G149E	P	[31]

© 2025 Xia Y et al. *JAMA Network Open.*

eTable 3 *SLC37A4* Variants in 113 GSDIb Patients (continued)

Patient ID	Paternal origin	Maternal origin	Allele 1 (paternal)				Allele 2 (maternal)			
			Nucleotide alteration (NM_001164277.2)	Amino acid alteration	Classification	ref.	Nucleotide alteration (NM_001164277.2)	Amino acid alteration	Classification	ref.
P18	Shandong	Guizhou	c.572C>T	p.P191L	P	27	c.822dupT	p.V275CfsX51	P	this study
P19	Guangdong	Guangdong	c.446G>A	p.G149E	P	31	c.446G>A	p.G149E	P	31
P20	Liaoning	Shaanxi	c.68T>G	p.L23R	LP	26	c.343G>A	p.G115R	P	26
P21	Shaanxi	Shaanxi	c.446G>A	p.G149E	P	25	c.446G>A	p.G149E	P	25
P22	Fujian	Fujian	c.191A>G	p.K64R	LP	this study	c.215A>C	p.D72A	P	this study
P23	Sichuan	Sichuan	c.359del	p.P120HfsX26	P	32	c.343G>A	p.G115R	P	26
P24	Jiangsu	Jiangsu	c.572C>T	p.P191L	P	27	c.11dupA	p.Y6LfsX43	P	this study
P25	Henan	Henan	c.1243C>T	p.R415X	P	26	c.68T>G	p.L23R	LP	26
P26	Heilongjiang	Shandong	c.288G>A	p.W96X	P	33	c.11dupA	p.Y6LfsX43	P	this study
P27	Jilin	Jilin	c.984+1G>A	/	P	ClinVar	c.752T>C	p.L251P	P	LOVD (#0000874563)
P28	Hubei	Hubei	c.1014_1120del	p.F338LfsX28	P	this study	c.310_311insT	p.A104VfsX7	P	this study
P29	Guangdong	Guangdong	c.446G>A	p.G149E	P	31	c.446G>A	p.G149E	P	31
P30	Hebei	Hebei	c.1042_1043del	p.L348VfsX53	P	24	c.352del	p.W118GfsX28	P	this study
P31	Fujian	Fujian	c.736T>C	p.W246R	LP	ClinVar	c.899G>A	p.R300H	P	26
P32	Guangdong	Guangdong	c.145T>G	p.L49V	LP	ClinVar	c.446G>A	p.G149E	P	31
P33	Guangdong	Guangdong	c.145T>G	p.L49V	LP	ClinVar	c.446G>A	p.G149E	P	31
P34	Guizhou	Guizhou	c.239T>C	p.F80S	LP	this study	c.359del	p.P120HfsX26	P	32
P35	Guangdong	Guangdong	c.446G>A	p.G149E	P	31	c.446G>A	p.G149E	P	31

© 2025 Xia Y et al. *JAMA Network Open.*

eTable 3 *SLC37A4* Variants in 113 GSDIb Patients (continued)

Patient ID	Paternal origin	Maternal origin	Allele 1 (paternal)				Allele 2 (maternal)			
			Nucleotide alteration (NM_001164277.2)	Amino acid alteration	Classification	ref.	Nucleotide alteration (NM_001164277.2)	Amino acid alteration	Classification	ref.
P36	Shandong	Heilongjiang	c.572C>T	p.P191L	P	[27]	c.752T>C	p.L251P	P	LOVD (#0000874563)
P37	Guangdong	Guangdong	c.446G>A	p.G149E	P	[31]	c.446G>A	p.G149E	P	[31]
P38	Guangxi	Guangxi	c.446G>A	p.G149E	P	[31]	c.572C>T	p.P191L	P	[27]
P39	Sichuan	Sichuan	c.1179G>A	p.W393X	P	[33]	c.239T>C	p.F80S	LP	this study
P40	Guangdong	Guangdong	c.870+5G>A	/	VUS	[26]	c.572C>T	p.P191L	P	[27]
P41	Zhejiang	Zhejiang	c.2del	/	VUS	this study	c.1065del	p.S356VfsX47	P	this study
P42	Guangdong	Guangdong	c.446G>A	p.G149E	P	[31]	c.446G>A	p.G149E	P	[31]
P43	Shanxi	Shanxi	c.572C>T	p.P191L	P	[27]	c.11dupA	p.Y6LfsX43	P	this study
P44	Guangdong	Guangdong	c.446G>A	p.G149E	P	[31]	c.446G>A	p.G149E	P	[31]
P45	Guangdong	Guangdong	c.446G>A	p.G149E	P	[31]	c.446G>A	p.G149E	P	[31]
P46	Guangdong	Guangdong	c.572C>T	p.P191L	P	[27]	c.446G>A	p.G149E	P	[31]
P47	Guangdong	Guangdong	c.572C>T	p.P191L	P	[27]	c.446G>A	p.G149E	P	[31]
P48	Shaanxi	Shaanxi	c.359C>G	p.P120R	VUS	this study	c.898C>T	p.R300C	LP	[26]
P49	Jiangsu	Jiangsu	c.154A>T	p.I52F	LP	this study	c.1108_1109del	p.G370TfsX31	P	ClinVar
P50	Sichuan	Sichuan	c.1042_1043del	p.L348VfsX53	P	[29]	c.343G>A	p.G115R	P	[26]
P51	Guangdong	Guangdong	c.736T>C	p.W246R	LP	ClinVar	c.572C>T	p.P191L	P	[27]
P52	Sichuan	Sichuan	c.572C>T	p.P191L	P	[27]	c.354G>A	p.W118X	P	this study
P53	Henan	Henan	c.1243C>T	p.R415X	P	[26]	c.68T>G	p.L23R	LP	[26]
P54	Guizhou	Guizhou	c.572C>T	p.P191L	P	[27]	c.572C>T	p.P191L	P	[27]

© 2025 Xia Y et al. *JAMA Network Open.*

eTable 3 *SLC37A4* Variants in 113 GSDIb Patients (continued)

Patient ID	Paternal origin	Maternal origin	Allele 1 (paternal)				Allele 2 (maternal)			
			Nucleotide alteration (NM_001164277.2)	Amino acid alteration	Classification	ref.	Nucleotide alteration (NM_001164277.2)	Amino acid alteration	Classification	ref.
P55	Shanxi	Hebei	c.755_756del	p.I252TfsX11	P	this study	c.361T>C	p.C121R	VUS	this study
P56	Hebei	Hebei	c.984+1G>A	/	P	ClinVar	c.752T>C	p.L251P	P	LOVD (#0000874563)
P57	Hunan	Hunan	c.359dupC	p.C121MfsX10	P	30	c.83G>A	p.R28H	LP	34
P58	Jiangsu	Jiangsu	c.1123+3_1123+6del	/	VUS	this study	c.215A>C	p.D72A	P	this study
P59	Shanghai	Shanghai	c.572C>T	p.P191L	P	27	c.788G>A	p.S263N	VUS	this study
P60	Yunnan	Yunnan	c.1109G>A	p.G370E	VUS	this study	c.572C>T	p.P191L	P	27
P61	Guangdong	Guangdong	c.446G>A	p.G149E	P	25	c.446G>A	p.G149E	P	25
P62	Guangdong	Guangdong	c.572C>T	p.P191L	P	27	c.446G>A	p.G149E	P	25
P63	Liaoning	Liaoning	c.11dupA	p.Y6LfsX43	P	this study	c.83G>A	p.R28H	LP	34
P64	Henan	Henan	c.572C>T	p.P191L	P	27	c.572C>T	p.P191L	P	27
P65	Shaanxi	Shaanxi	c.572C>T	p.P191L	P	27	c.343G>A	p.G115R	P	26
P66	Jiangxi	Jiangxi	c.446G>A	p.G149E	P	25	c.55G>C	p.G19R	LP	this study
P67	Jilin	Hubei	c.572C>T	p.P191L	P	27	c.572C>T	p.P191L	P	27
P68	Guangdong	Guangdong	c.446G>A	p.G149E	P	25	c.446G>A	p.G149E	P	25
P69	Jiangsu	Jiangsu	c.1243C>T	p.R415X	P	26	c.1042_1043del	p.L348VfsX53	P	29
P70	Hebei	Hebei	c.1177T>C	p.W393R	VUS	this study	c.1042_1043del	p.L348VfsX53	P	29
P71	Guangdong	Guangdong	c.446G>A	p.G149E	P	31	c.446G>A	p.G149E	P	31

© 2025 Xia Y et al. *JAMA Network Open.*

eTable 3 *SLC37A4* Variants in 113 GSDIb Patients (continued)

Patient ID	Paternal origin	Maternal origin	Allele 1 (paternal)				Allele 2 (maternal)			
			Nucleotide alteration (NM_001164277 .2)	Amino acid alteration	Classifi cation	ref.	Nucleotide alteration (NM_001164 277.2)	Amino acid alteration	Classifi cation	ref.
P72	Guangdong	Guangdong	c.446G>A	p.G149E	P	[31]	c.446G>A	p.G149E	P	[31]
P73	Guangdong	Guangdong	c.343G>A	p.G115R	P	[26]	c.446G>A	p.G149E	P	[31]
P74	Shanxi	Shanxi	c.572C>T	p.P191L	P	[27]	c.1043T>C	p.L348P	LP	[27]
P75	Guangdong	Guangdong	c.446G>A	p.G149E	P	[25]	c.59G>A	p.G20D	LP	[35]
P76	Hebei	Hebei	c.1079_1097del	p.N360TfsX37	P	this study	c.572C>T	p.P191L	P	[27]
P77	Shanxi	Shanxi	c.572C>T	p.P191L	P	[27]	c.572C>T	p.P191L	P	[27]
P78	Shanxi	Shanxi	c.572C>T	p.P191L	P	[27]	c.572C>T	p.P191L	P	[27]
P79	Jiangsu	Jiangsu	c.1042_1043del	p.L348VfsX53	P	[29]	c.1042_1043 del	p.L348VfsX53	P	[29]
P80	Hubei	Hubei	c.572C>T	p.P191L	P	[27]	c.1179G>A	p.W393X	P	[36]
P81	Hubei	Hubei	c.572C>T	p.P191L	P	[27]	c.1179G>A	p.W393X	P	[36]
P82	Guangdong	Guangdong	c.572C>T	p.P191L	P	[27]	c.343G>A	p.G115R	P	[26]
P83	Shanxi	Shanxi	c.572C>T	p.P191L	P	[27]	c.572C>T	p.P191L	P	[27]
P84	Guangdong	Guangdong	c.446G>A	p.G149E	P	[31]	c.343G>A	p.G115R	P	[26]
P85	Hunan	Shaanxi	c.572C>T	p.P191L	P	[27]	c.446G>A	p.G149E	P	[31]
P86	Guangxi	Guangxi	c.446G>A	p.G149E	P	[31]	c.446G>A	p.G149E	P	[31]
P87	Guangdong	Guangdong	c.446G>A	p.G149E	P	[31]	c.446G>A	p.G149E	P	[31]
P88	Jiangsu	Jiangsu	c.576dupT	p.D193X	P	this study	c.257T>G	p.L86R	VUS	this study
P89	Jilin	Zhejiang	c.321G>A	p.W107X	P	[31]	c.446G>A	p.G149E	P	[31]
P90	Jiangsu	Jiangsu	c.1123+3_1123+ 6del	/	VUS	this study	c.1042_1043 del	p.L348VfsX53	P	[29]

© 2025 Xia Y et al. *JAMA Network Open.*

eTable 3 *SLC37A4* Variants in 113 GSDIb Patients (continued)

Patient ID	Paternal origin	Maternal origin	Allele 1 (paternal)				Allele 2 (maternal)			
			Nucleotide alteration (NM_001164277.2)	Amino acid alteration	Classification	ref.	Nucleotide alteration (NM_001164277.2)	Amino acid alteration	Classification	ref.
P91	Hunan	Hunan	c.359dupC	p.C121MfsX10	P	[30]	c.321G>A	p.W107X	P	[31]
P92	Hunan	Hunan	c.706_708del	p.V236del	LP	this study	c.1042_1043del	p.L348VfsX53	P	[29]
P93	Guangdong	Guangxi	c.119_120insC	p.E40DfsX9	P	this study	c.446G>A	p.G149E	P	[31]
P94	Fujian	Fujian	c.191A>G	p.K64R	LP	this study	c.736T>C	p.W246R	LP	ClinVar
P95	Sichuan	Sichuan	c.1179G>A	p.W393X	P	[36]	c.870+5G>A	/	VUS	[26]
P96	Guangdong	Guangdong	c.572C>T	p.P191L	P	[27]	c.446G>A	p.G149E	P	[31]
P97	Hubei	Anhui	c.1042_1043del	p.L348VfsX53	P	[29]	c.85A>C	p.K29Q	LP	this study
P98	Hunan	Guangdong	c.125_126insGA	p.I42MfsX34	P	this study	c.446G>A	p.G149E	P	[31]
P99	Chongqing	Chongqing	c.1179G>A	p.W393X	P	[36]	c.11dupA	p.Y6LfsX43	P	this study
P100	Chongqing	Chongqing	c.1179G>A	p.W393X	P	[36]	c.11dupA	p.Y6LfsX43	P	this study
P101	Shaanxi	Shaanxi	c.215A>C	p.D72A	P	this study	c.1042_1043del	p.L348VfsX53	P	[29]
P102	Shandong	Shandong	c.343G>A	p.G115R	P	[26]	c.343G>A	p.G115R	P	[26]
P103	Anhui	Anhui	c.1127del	p.G376AfsX27	P	this study	c.1070_1087del	p.A357_C362del	LP	this study
P104	Zhejiang	Zhejiang	c.215A>C	p.D72A	P	this study	c.288G>A	p.W96X	P	[33]
P105	Shaanxi	Shaanxi	c.62A>C	p.Y21S	LP	this study	c.359dupC	p.C121MfsX10	P	[30]
P106	Jiangxi	Jiangxi	c.446G>A	p.G149E	P	[31]	c.359dupC	p.C121MfsX10	P	[30]
P107	Jiangsu	Jiangsu	c.161G>A	p.S54N	LP	this study	c.215A>C	p.D72A	P	this study
P108	Guangdong	Guangdong	c.446G>A	p.G149E	P	[31]	c.446G>A	p.G149E	P	[31]

© 2025 Xia Y et al. *JAMA Network Open.*

eTable 3 *SLC37A4* Variants in 113 GSDIb Patients (continued)

Patient ID	Paternal origin	Maternal origin	Allele 1 (paternal)				Allele 2 (maternal)			
			**Nucleotide alteration (NM_001164277.2)**	**Amino acid alteration**	**Classification**	**ref.**	**Nucleotide alteration (NM_001164277.2)**	**Amino acid alteration**	**Classification**	**ref.**
P109	Zhejiang	Hunan	c.343G>A	p.G115R	P	[26]	c.92_94del	p.F31del	LP	this study
P110	Shanxi	Shanxi	c.359C>T	p.P120L	VUS	this study	c.752T>C	p.L251P	P	LOVD (#0000874563)
P111	Guangxi	Guangxi	c.795C>G	p.Y265X	P	this study	c.446G>A	p.G149E	P	[31]
P112	Guangdong	Guangdong	c.343G>A	p.G115R	P	[26]	c.1063G>T	p.E355X	LP	[37]
P113	Guangdong	Guangdong	c.343G>A	p.G115R	P	[26]	c.1063G>T	p.E355X	LP	[37]

/: unknown amino acid alteration; LP: likely pathogenic; P: pathogenic; VUS: variant of unknown significance. The cohort included six pairs of siblings with GSDIb: P32 and P33, P46 and P47, P77 and P78, P80 and P81, P99 and P100, P112 and P113.

© 2025 Xia Y et al. *JAMA Network Open.*

eTable 4 Laboratory Data at Baseline and After UCCS Treatment of 113 GSDIb Patients

	At baseline					After UCCS treatment	*Baseline vs treatment*	*Intergroup comparison**
	**≤ 2.0 years (n = 82)**	**2.1-5.0 years (n = 16)**	**5.1-10.0 years (n = 8)**	**> 10.0 years (n = 7)**	**Overall (n = 113)**	**Overall (n = 96)**		
**Laboratory data**	**Median (IQR)**							
Lactate, mg/dL	75.14 (54.50-102.70) (n = 68)	89.46 (67.84-112.88) (n = 14)	72.52 (43.42-97.48) (n = 6)	40.36 (33.78-53.60) (n = 6)	75.23 (52.61-102.70) (n = 94)	29.73 (19.82-44.59) (n =80)	*P* < .001	*P* = .01
ALT, U/L	107.0 (77.8-141.3) (n = 74)	93.5 (68.0-181.5) (n = 14)	61.0 (19.0-117.0) (n = 7)	54.5 (18.3-103.6) (n = 6)	97.0 (69.7-139.0) (n = 101)	18.0 (11.3-30.3) (n = 96)	*P* < .001	*P* = .03
AST, U/L	178.5 (130.8-251.5) (n =74)	131.0 (76.9-284.3) (n = 14)	73.0 (20.0-99.0) (n = 7)	64.0 (21.5-133.0) (n = 6)	156.0 (108.5-234.0) (n = 101)	25.0 (16.3-37.5) (n = 96)	*P* < .001	*P* < .001
GGT, U/L	220.0 (152.0-401.5) (n = 74)	139.5 (108.3-207.5) (n = 14)	79.0 (25.0-88.0) (n = 7)	69.5 (30.3-131.7) (n = 6)	191.0 (102.5-326.5) (n = 101)	25.0 (19.3-50.8) (n = 96)	*P* < .001	*P* < .001
TC, mg/dL	167.18 (136.29-233.98) (n = 66)	206.95 (177.22-281.47) (n = 12)	150.58 (131.27-171.43) (n = 7)	162.93 (120.46-252.90) (n = 6)	170.66 (138.61-233.20) (n = 91)	139.77 (118.15-182.24) (n =96)	*P* < .001	*P* = .07
TG, mg/dL	600.00 (400.00-937.17) (n = 66)	485.84 (343.36-786.73) (n = 12)	315.93 (224.78-415.04) (n = 7)	757.52 (501.77-864.60) (n = 6)	564.60 (372.57-824.78) (n = 91)	189.38 (120.35-268.14) (n = 96)	*P* < .001	*P* = .03
UA z score	4.73 (2.65-7.08) (n = 72)	6.02 (3.73-9.17) (n = 14)	3.19 (1.34-4.69) (n = 7)	3.65 (0.05-6.25) (n = 6)	4.69 (2.64-6.88) (n = 99)	3.34 (1.84-5.22) (n =96)	*P* = .003	*P* = .13

© 2025 Xia Y et al. *JAMA Network Open.*

eTable 4 Laboratory Data at Baseline and After UCCS Treatment of 113 GSDIb Patients (continued)

	At baseline					After UCCS treatment	*Baseline vs treatment*	*Intergroup comparison**
	≤ 2.0 years (n = 82)	2.1-5.0 years (n = 16)	5.1-10.0 years (n = 8)	> 10.0 years (n = 7)	Overall (n = 113)	Overall (n = 96)		
**Laboratory data**	**Percentage (No., %)**							
Neutropenia	53/72 (74)	12/14 (86)	7/7 (100)	5/6 (83)	77 of 99 (78)	74 of 96 (77)	*P* > .99	*P* = .34
Severe neutropenia	14/72 (19)	5/14 (36)	2/7 (29)	3/6 (50)	24 of 99 (24)	31 of 96 (32)	*P* = .27	*P* = .25
Anemia	48/72 (67)	9/14 (64)	4/7 (57)	5/6 (83)	66 of 99 (67)	54 of 96 (56)	*P* = .14	*P* = .78

UCCS: uncooked cornstarch; IQR: interquartile range; Conversion factor for corresponding SI units: ALT (alanine aminotransferase) 0.0167 for µkat/L; AST (aspartate aminotransferase) 0.0167 for µkat/L; GGT (γ-glutamyl transpeptidase) 0.0167 for µkat/L; lactate 0.111 for mmol/L; TC (total cholesterol) 0.0259 for mmol/L; TG (triglycerides) 0.0113 for mmol/L; UA: uric acid. *: examining variations in laboratory parameter levels across four age groups at baseline.

© 2025 Xia Y et al. *JAMA Network Open.*

eTable 5 Details of Arthritis in Six GSDIb Patients

Patient ID	Sex	Onset age of arthritis (years)	Number of episodes (times)	Duration of each episode (months)	Site
P9	M	18.0	6	1	Bilateral ankles, left knee
P12	M	5.0	6	4	Bilateral knees, right shoulder
P30	M	6.0	3	1	Bilateral knees
P36	M	7.0	NA	Persistent except during periods of intermittent corticosteroid use	Bilateral hips, knees and ankles
P39	M	14.0	2	6	Bilateral shoulders
P88	M	24.5	1	5	Bilateral hips, knees and shoulders

M: male; NA: not applicable.

© 2025 Xia Y et al. *JAMA Network Open.*

eTable 6 Mean ΔHeight z Scores per-Year Interval at Baseline and After UCCS Treatment in GSDIb Patients

Age (years)	At baseline			After UCCS treatment			*Baseline vs treatment*
	Mean	Standard deviation	n	Mean	Standard deviation	n	
1	-1.47	1.53	101	-0.99	1.22	15	*P* = .25
2	-1.96	0.97	31	-1.14	1.07	41	*P* = .001
3	-2.68	0.96	12	-1.65	1.39	35	*P* = .02
4	-2.57	1.4	7	-1.49	1.43	20	*P* = .10
5	-3.41	1.11	5	-1.21	1.85	14	*P* = .02
6	-3.71	1.66	3	-2.44	1.5	14	*P* = .21
7	-3.27	0.96	4	-2.75	1.4	16	*P* = .49
8	NA	NA	NA	-2.54	1.67	13	NA
9	-3.34	NA	1	-2.81	1.34	11	NA
10	-3.29	NA	1	-2.86	1.24	11	NA
11	NA	NA	NA	-2.88	0.88	6	NA
12	-4.35	NA	2	-3.38	1.56	12	NA
13	-4.39	NA	2	-3.93	0.83	6	NA
14	-4.38	NA	2	-4.21	1.32	11	NA
15	NA	NA	NA	-3.39	2.18	7	NA
16	NA	NA	NA	-3.24	3.55	4	NA
17	-3.48	NA	1	-2.94	0.84	3	NA
18	-3.4	NA	2	-2.7	1.18	12	NA

NA: data missing or not applicable; UCCS: uncooked cornstarch.

© 2025 Xia Y et al. *JAMA Network Open.*

eTable 7 Clinical Changes in P56 Before and After Liver Transplant

Presentations and laboratory parameters	Before	After
Frequency of respiratory tract infections (times/year)	12	6
Frequency of oral ulcers (times/year)	24	5
Diarrhea	Y	N
Abdominal pain	Y	N
Perianal abscess	Y	N
White blood cell (μL) (reference range: 4000 ~ 10000)	4240	2980
Absolute neutrophile count (μL) (reference range: ≥ 1500)	290	490
Pediatric Crohn's Disease Activity Index (reference range: < 10)	72.5	22.5
Δ Height z score (reference range: > -2)	-3.11	-0.76
BMI z score (reference range: 2 ~ -2)	-1.42	-0.05

Y: yes; N: no; BMI: body mass index.

© 2025 Xia Y et al. *JAMA Network Open.*

eTable 8 Details of Deceased GSDIb Patients

Patient ID	Year of birth	Age of death (years)	Cause of death	G-CSF/IBD treatment
P68	2004	7.2	MD, severe pneumonia, sepsis	No
P86	1989	27.4	IBD	No
P88	1997	25.5	MD, pulmonary hypertension, heart failure	Yes
P89	1998	17.0	Car accident	No
P102	2005	0.7	MD, severe pneumonia, sepsis	No
P103	2020	0.5	MD, severe pneumonia, sepsis, convulsive status epilepticus	No
P104	2016	0.4	MD, severe pneumonia, sepsis	No
P105	2014	7.0	MD, severe pneumonia, sepsis	No
P109	2017	2.1	MD, severe pneumonia, sepsis	No
P111	2011	1.6	MD, sepsis	No
P113	2023	0.1	MD, sepsis, neonatal necrotizing enterocolitis, congenital heart disease, premature birth	No

MD: metabolic derangements; IBD: inflammatory bowel disease; G-CSF: granulocyte colony-stimulating factor.

© 2025 Xia Y et al. *JAMA Network Open.*

eTable 9 Details of Five Dropout GSDIb Patients

Patient ID	Sex	Age at the final visit (years)	Reasons for dropping out	G-CSF	IBD treatment	Possible familial factors for dropping out
P2	F	13.6	Low intelligence	N	N	Low income, rural resident
P20	F	13.8	Severe IBD	Y	Y	NA
P68	M	7.2	Recurrent infections	N	N	Low income, rural resident
P86	F	27.4	Severe IBD	N	N	Low income, rural resident, multi-child family
P88	M	25.5	Severe IBD	Y	Y	NA

IBD: inflammatory bowel disease; F: female; M: male; G-CSF: granulocyte colony-stimulating factor; Y: yes; N: no; NA: not applicable.

© 2025 Xia Y et al. *JAMA Network Open.*

eFigure 1 Geographical Distribution of Patients' origins (a), p.G149E (b) and p.P191L (c) in China

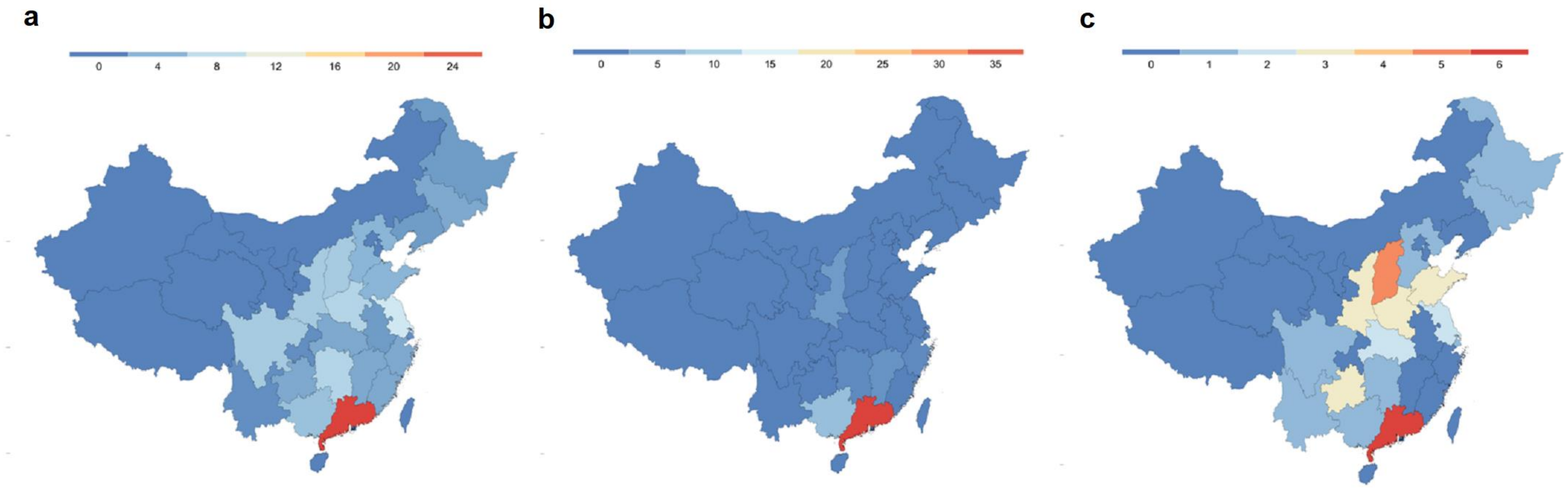

The redder the color of a province, the more the patient or variant allele from this province. The bluer the color of a province, the fewer the patient or variant allele from this province.

© 2025 Xia Y et al. *JAMA Network Open.*

eFigure 2 Biochemical Control of GSDIb Cohort

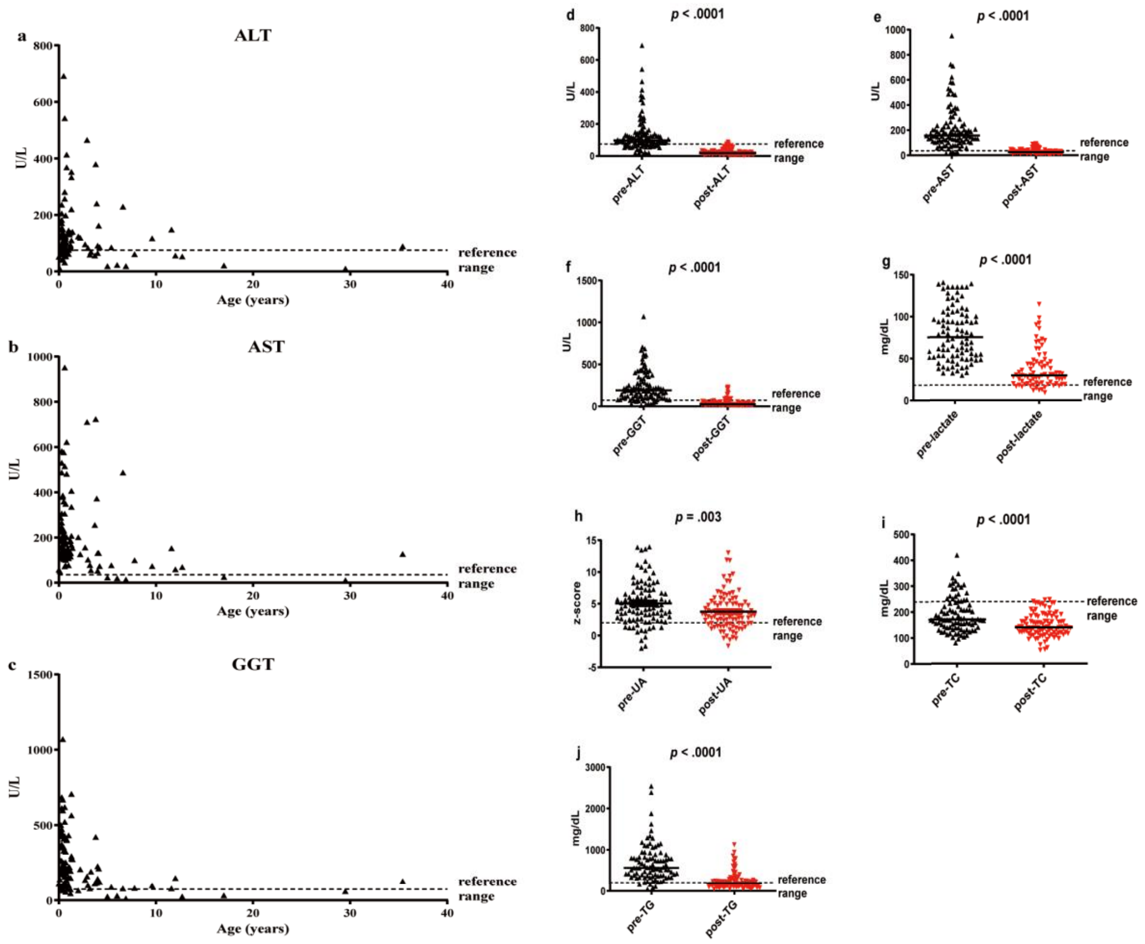

The levels of alanine aminotransferase (ALT, a, d), aspartate aminotransferase (AST, b, e), γ-glutamyl transpeptidase (GGT, c, f), lactate (g), uric acid (UA) z score (h), total cholesterol (TC, i) and triglyceride (TG, j) of patients at baseline (pre) and after uncooked cornstarch (UCCS) treatment (post) are illustrated as black and red triangles, respectively, with reference ranges for each parameter indicated by dashed lines.

© 2025 Xia Y et al. *JAMA Network Open.*

eReferences

1. Kishnani PS, Austin SL, Abdenur JE, et al. Diagnosis and management of glycogen storage disease type I: a practice guideline of the American College of Medical Genetics and Genomics. *Genet Med*. Nov 2014;16(11):e1. doi:10.1038/gim.2014.128

2. Thornton PS, Stanley CA, De Leon DD, et al. Recommendations from the Pediatric Endocrine Society for Evaluation and Management of Persistent Hypoglycemia in Neonates, Infants, and Children. *J Pediatr*. Aug 2015;167(2):238-45. doi:10.1016/j.jpeds.2015.03.057

3. Foucher CD, Tubben RE. Lactic Acidosis. *StatPearls*. 2024.

4. Allergy Group of Chinese Pediatric Doctor Association RGoCPDA, Rheumatology Immunology Group of Chinese Pediatric Doctor Association. Clinical diagnosis and treatment pathway of recurrent respiratory tract infection in children (2022). *Chinese Journal of Practical Pediatrics*. 2022;037-003

5. Ibler KS, Kromann CB. Recurrent furunculosis - challenges and management: a review. *Clin Cosmet Investig Dermatol*. 2014;7:59-64. doi:10.2147/CCID.S35302

6. Nemeth V, Pfleghaar N. Diarrhea. *StatPearls*. 2024.

7. Katsanis E, Luke KH, Hsu E, Li M, Lillicrap D. Prevalence and significance of mild bleeding disorders in children with recurrent epistaxis. *J Pediatr*. Jul 1988;113(1 Pt 1):73-6. doi:10.1016/s0022-3476(88)80532-8

© 2025 Xia Y et al. *JAMA Network Open.*

8. Li H, Ji CY, Zong XN, Zhang YQ. [Height and weight standardized growth charts for Chinese children and adolescents aged 0 to 18 years]. *Zhonghua Er Ke Za Zhi*. Jul 2009;47(7):487-92.

9. Coordinating Study Group of Nine Cities on Physical G, Development of C, Capital Institute of P. [A national survey on growth of children under 7 years of age in nine cities of China, 2005]. *Zhonghua Er Ke Za Zhi*. Aug 2007;45(8):609-14.

10. Joint Expert Committee on Revision of Chinese Guidelines for Blood Lipid Management LJ, Zhao Weng, Gao Runlin. Chinese Guidelines for Blood Lipid Management (2023). *Chinese Journal of Circulation*. 2023;38(3):237-271.

11. Kubota M. Hyperuricemia in Children and Adolescents: Present Knowledge and Future Directions. *J Nutr Metab*. 2019;2019:3480718. doi:10.1155/2019/3480718

12. Donadieu J, Fenneteau O, Beaupain B, Mahlaoui N, Chantelot CB. Congenital neutropenia: diagnosis, molecular bases and patient management. *Orphanet J Rare Dis*. May 19 2011;6:26. doi:10.1186/1750-1172-6-26

13. Lv Y, Feng G, Ni X, Song W, Peng X. The critical gap for pediatric reference intervals of complete blood count in China. *Clin Chim Acta*. Jun 2017;469:22-25. doi:10.1016/j.cca.2017.03.017

14. Bidlingmaier M, Friedrich N, Emeny RT, et al. Reference intervals for insulin-like growth factor-1 (igf-i) from birth to senescence: results from a

© 2025 Xia Y et al. *JAMA Network Open.*

multicenter study using a new automated chemiluminescence IGF-I immunoassay conforming to recent international recommendations. *J Clin Endocrinol Metab*. May 2014;99(5):1712-21. doi:10.1210/jc.2013-3059

15. Stevens PE, Levin A, Kidney Disease: Improving Global Outcomes Chronic Kidney Disease Guideline Development Work Group M. Evaluation and management of chronic kidney disease: synopsis of the kidney disease: improving global outcomes 2012 clinical practice guideline. *Ann Intern Med*. Jun 4 2013;158(11):825-30. doi:10.7326/0003-4819-158-11-201306040-00007

16. Levey AS, de Jong PE, Coresh J, et al. The definition, classification, and prognosis of chronic kidney disease: a KDIGO Controversies Conference report. *Kidney Int*. Jul 2011;80(1):17-28. doi:10.1038/ki.2010.483

17. Liu J, Wang L, Sun J, et al. Bone mineral density reference standards for Chinese children aged 3-18: cross-sectional results of the 2013-2015 China Child and Adolescent Cardiovascular Health (CCACH) Study. *BMJ Open*. May 29 2017;7(5):e014542. doi:10.1136/bmjopen-2016-014542

18. Levey AS, Stevens LA, Schmid CH, et al. A new equation to estimate glomerular filtration rate. *Ann Intern Med*. May 5 2009;150(9):604-12. doi:10.7326/0003-4819-150-9-200905050-00006

19. Schwartz GJ, Work DF. Measurement and estimation of GFR in children and adolescents. *Clin J Am Soc Nephrol*. Nov 2009;4(11):1832-43. doi:10.2215/CJN.01640309

© 2025 Xia Y et al. *JAMA Network Open.*

20. Maaser C, Sturm A, Vavricka SR, et al. ECCO-ESGAR Guideline for Diagnostic Assessment in IBD Part 1: Initial diagnosis, monitoring of known IBD, detection of complications. *J Crohns Colitis*. Feb 1 2019;13(2):144-164. doi:10.1093/ecco-jcc/jjy113

21. Dhaliwal J, Walters TD, Mack DR, et al. Phenotypic Variation in Paediatric Inflammatory Bowel Disease by Age: A Multicentre Prospective Inception Cohort Study of the Canadian Children IBD Network. *J Crohns Colitis*. May 21 2020;14(4):445-454. doi:10.1093/ecco-jcc/jjz106

22. Best WR, Becktel JM, Singleton JW, Kern F, Jr. Development of a Crohn's disease activity index. National Cooperative Crohn's Disease Study. *Gastroenterology*. Mar 1976;70(3):439-44.

23. Dassopoulos T, Nguyen GC, Bitton A, et al. Assessment of reliability and validity of IBD phenotyping within the National Institutes of Diabetes and Digestive and Kidney Diseases (NIDDK) IBD Genetics Consortium (IBDGC). *Inflamm Bowel Dis*. Aug 2007;13(8):975-83. doi:10.1002/ibd.20144

24. Choi R, Park HD, Ko JM, et al. Novel SLC37A4 Mutations in Korean Patients With Glycogen Storage Disease Ib. *Ann Lab Med*. May 2017;37(3):261-266. doi:10.3343/alm.2017.37.3.261

25. Sperb-Ludwig F, Pinheiro FC, Bettio Soares M, et al. Glycogen storage diseases: Twenty-seven new variants in a cohort of 125 patients. *Mol Genet Genomic Med*. Nov 2019;7(11):e877. doi:10.1002/mgg3.877

26. Chou JY, Mansfield BC. The SLC37 family of sugar-phosphate/phosphate exchangers. *Curr Top Membr*. 2014;73:357-82. doi:10.1016/B978-0-12-

© 2025 Xia Y et al. *JAMA Network Open.*

800223-0.00010-4

27. Wang J, Cui H, Lee NC, et al. Clinical application of massively parallel sequencing in the molecular diagnosis of glycogen storage diseases of genetically heterogeneous origin. *Genet Med*. Feb 2013;15(2):106-14. doi:10.1038/gim.2012.104

28. Yuen YP, Cheng WF, Tong SF, Chan YT, Chan YW, Lam CW. Novel missense mutation (Y24H) in the G6PT1 gene causing glycogen storage disease type 1b. *Mol Genet Metab*. Nov 2002;77(3):249-51. doi:10.1016/s1096-7192(02)00110-5

29. Bali DS, El-Gharbawy A, Austin S, Pendyal S, Kishnani PS. Glycogen Storage Disease Type I. In: Adam MP, Feldman J, Mirzaa GM, et al, eds. *GeneReviews((R))*. 1993.

30. Veiga-da-Cunha M, Gerin I, Chen YT, et al. The putative glucose 6-phosphate translocase gene is mutated in essentially all cases of glycogen storage disease type I non-a. *Eur J Hum Genet*. Sep 1999;7(6):717-23. doi:10.1038/sj.ejhg.5200366

31. Lee KJ, Choi SJ, Kim WS, Park SS, Moon JS, Ko JS. Esophageal Stricture Secondary to Candidiasis in a Child with Glycogen Storage Disease 1b. *Pediatr Gastroenterol Hepatol Nutr*. Mar 2016;19(1):71-5. doi:10.5223/pghn.2016.19.1.71

32. Shao YX, Liang CL, Su YY, et al. Clinical spectrum, over 12-year follow-up and experience of SGLT2 inhibitors treatment on patients with glycogen storage disease type Ib: a single-center retrospective study. Orphanet J Rare Dis. Apr 11 2024;19(1):155. doi:10.1186/s13023-024-03137-6

© 2025 Xia Y et al. *JAMA Network Open.*

33. Melis D, Fulceri R, Parenti G, et al. Genotype/phenotype correlation in glycogen storage disease type 1b: a multicentre study and review of the literature. *Eur J Pediatr*. Aug 2005;164(8):501-8. doi:10.1007/s00431-005-1657-4

34. Hiraiwa H, Pan CJ, Lin B, Moses SW, Chou JY. Inactivation of the glucose 6-phosphate transporter causes glycogen storage disease type 1b. *J Biol Chem*. Feb 26 1999;274(9):5532-6. doi:10.1074/jbc.274.9.5532

35. Veiga-da-Cunha M, Gerin I, Chen YT, et al. A gene on chromosome 11q23 coding for a putative glucose- 6-phosphate translocase is mutated in glycogen-storage disease types Ib and Ic. *Am J Hum Genet*. Oct 1998;63(4):976-83. doi:10.1086/302068

36. Prasad R, Estrella J, Christodoulou J, McKellar G, Tchan MC. A Third Case of Glycogen Storage Disease IB and Giant Cell Tumour of the Mandible: A Disease Association or Iatrogenic Complication of Therapy. *JIMD Rep*. 2018;42:5-8. doi:10.1007/8904_2017_67

37. Chou JY, Matern D, Mansfield BC, Chen YT. Type I glycogen storage diseases: disorders of the glucose-6-phosphatase complex. *Curr Mol Med*. Mar 2002;2(2):121-43. doi:10.2174/1566524024605798

38. Sipponen T, Nuutinen H, Turunen U, Farkkila M. Endoscopic evaluation of Crohn's disease activity: comparison of the CDEIS and the SES-CD. *Inflamm Bowel Dis*. Dec 2010;16(12):2131-6. doi:10.1002/ibd.21300

© 2025 Xia Y et al. *JAMA Network Open.*
