# Supplementary material for: Genetic Variants and Clinical Features of Patients With Glycogen Storage Disease Type Ib
Source: JAMA Netw Open. 2025 Feb 26;8(2):e2461888. doi: 10.1001/jamanetworkopen.2024.61888 (PMC11866026; doi:10.1001/jamanetworkopen.2024.61888)
Supplement: Supplement 3. — Data Sharing Statement [file jamanetwopen-e2461888-s003.pdf]

Data Sharing Statement

Xia. Genetic Variants and Clinical Features of Patients With Glycogen Storage Disease Type Ib. *JAMA Netw Open*. Published February 26, 2025.
doi:10.1001/jamanetworkopen.2024.61888

Data
Data available: Yes
Data types: Deidentified participant data
How to access data: The data in the current study are available from the corresponding author (qiuwenjuan@xinhuamed.com.cn) upon reasonable request.
When available: With publication

Supporting Documents
Document types: None

Additional Information
Who can access the data: researchers whose proposed use of the data has been approved
Types of analyses: for any purpose
Mechanisms of data availability: with a signed data access agreement
